# Post-infectious inflammatory response syndrome related to cryptococcal meningoencephalitis

**DOI:** 10.1590/0037-8682-0438-2023

**Published:** 2023-10-13

**Authors:** João Luiz Veloso Mourão, Alessa Andrade Santana, Marcelo de Carvalho Ramos, Lucieni Conterno, Fabiano Reis

**Affiliations:** 1 Universidade Estadual de Campinas, Faculdade de Ciências Médicas, Departamento de Anestesiologia, Oncologia e Radiologia, Campinas, SP, Brasil.; 2 Universidade Estadual de Campinas, Faculdade de Ciências Médicas, Departamento de Medicina Interna, Campinas, SP, Brasil.

**FIGURE 1:**
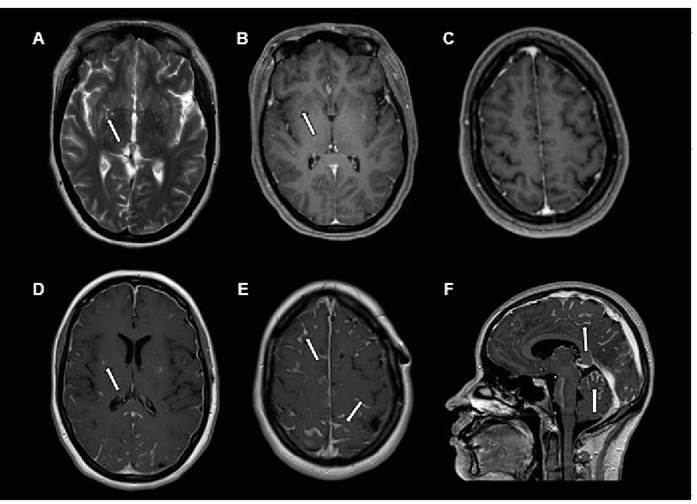
Magnetic resonance imaging axial T2-weighted image showing enlargement of the perivascular spaces in the nucleocapsular regions (arrows) **(A)**. Axial contrast-enhanced T1-weighted image showing minimal enhancement in the enlarged perivascular spaces (arrows) **(B)**. Axial contrast-enhanced T1 image on high convexity, with no enhancement of leptomeningitis **(C)**. Axial post-contrast T1-weighted image showing persistent enhancement of the enlarged perivascular spaces; note the marked leptomeningeal enhancement that is more conspicuous in the bilateral parieto-occipital region **(D and E)**. Sagittal post-contrast-enhanced T1-weighted image showing marked leptomeningeal enhancement in the frontal, parietal, and occipital lobes in the supratentorial region and also between the cerebellar folia in the infratentorial region, with the presence of leptomeningitis **(F)**.

A 24-year-old immunocompetent and previously healthy female was admitted to our Emergency Department with severe headaches, nausea, and vomiting. Brain magnetic resonance imaging (MRI) revealed bilateral dilation of the perivascular region (Figure 1A-C). Cerebrospinal fluid (CSF) culture was positive for *Cryptococcus gatti*. Liposomal amphotericin B (5 mg/kg/day) and fluconazole (1200 mg/day) were administered. A ventriculoperitoneal shunt was created to alleviate the intracranial hypertension. After improvement and a negative CSF culture for fungi, the patient was discharged with oral fluconazole (900 mg/day).

**FIGURE 2:**
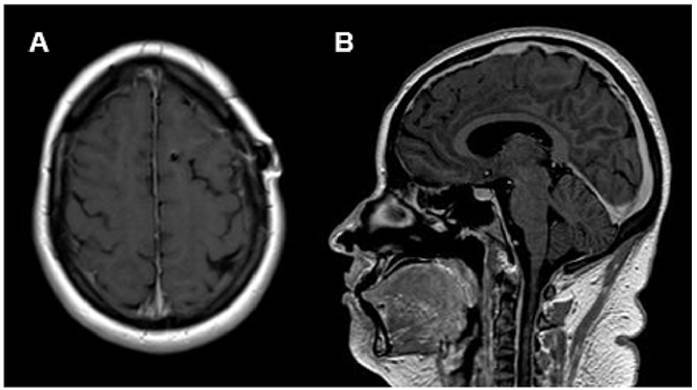
Axial **(A)** and sagittal **(B)** post-contrast T1-weighted images after the institution of immunosuppressive treatment. Note the absence of radiological evidence for leptomeningeal enhancement.

Twenty-eight days after discharge, the patient returned with complains of vomiting and severe headache. A new brain MRI revealed progression of the infectious condition with leptomeningitis (Figure 1D-F), although the CSF culture for fungi was negative. Despite treatment with amphotericin B (5 mg/kg/day) and flucytosine (1500 mg, four times a day), the patient showed no significant improvement. In view of this lack of improvement, the hypothesis of post-infectious inflammatory response syndrome (PIIRS) was proposed, and corticosteroid therapy (dexamethasone, an attack dose of 10 mg and a maintenance dose of 4 mg four times a day) was initiated, with significant improvement in the clinical and MRI findings (Figure 2A,B).

After treatment for neurocryptococcosis, immunocompetent patients may experience a relapse of inflammatory manifestations and leptomeningitis (leptomeningeal thickening and enhancement; Figure 1E,F) despite appropriate antifungal treatment^1^ ^,^ ^2^. This case reinforces the need for medical teams to be aware of this complication in immunocompetent patients who are undergoing treatment for neurocryptococcosis. Immunosuppressive therapy should be considered when culture results are negative^3^.
